# How I Treat: Immune dysregulation in Down syndrome

**DOI:** 10.70962/jhi.20250161

**Published:** 2026-05-04

**Authors:** Melissa Gans, Matthew Wyke, Kayra Somay, Willa Thorson, Dusan Bogunovic

**Affiliations:** 1 Department of Pediatrics, Division of Allergy & Immunology, Jackson Memorial Health System, University of Miami Miller School of Medicine, Miami, FL, USA; 2 Department of Pediatrics, Division of Genetics, Jackson Memorial Health System, University of Miami Miller School of Medicine, Miami, FL, USA; 3 Department of Pediatrics, Columbia Center for Genetic Errors of Immunity, Columbia University Irving Medical Center, New York, NY, USA

## Abstract

Immune dysregulation in Down syndrome (DS) is a highly clinically relevant yet underrecognized feature of this common genetic syndrome. Infection is the leading cause of mortality in DS. This infectious risk likely stems from underlying immunodeficiency compounded by their unique intrinsic anatomy. Furthermore, individuals with DS display a broad range of autoinflammatory and autoimmune diseases, spanning dermatologic, neurologic, and metabolic features, which are often difficult to treat with immunomodulators. Through real clinical vignettes of our patients, we will describe how we treat immune dysregulation in DS.

## Teaser

Immune dysregulation is a common yet underrecognized feature of Down syndrome that contributes to infections, autoimmunity, and autoinflammation. Using clinical vignettes, this work highlights the underlying mechanisms and potential treatments for the immune dysregulation in Down syndrome.

## Introduction

Down syndrome (DS) is the most common chromosomal abnormality found in live births, with an estimation of 5,000 babies born per year with DS in the United States (1). Owing to significant medical advances, individuals with DS now routinely achieve longer lifespans and live well into adulthood (2). While this condition is familiar to many physicians and carries many known comorbidities, immune dysregulation is one of the least recognized yet clinically relevant features of the disease. Patients are predisposed to severe infection, autoimmunity, and autoinflammation due to several underlying mechanisms of which evidence has been compounding over recent years (3, 4, 5, 6, 7, 8). Thymic hypoplasia, innate and adaptive cell defects, and abnormal cytokine and interferon (IFN) signaling are some of the mechanisms underlying this immune dysfunction (9, 10, 11, 12). This is of significant relevance given the comorbid disease states seen in DS that increase the risk of severe infectious or inflammatory morbidity such as thymus-removing congenital heart surgery, upper airway anatomical defects, and hypotonia.

**Figure 1.**
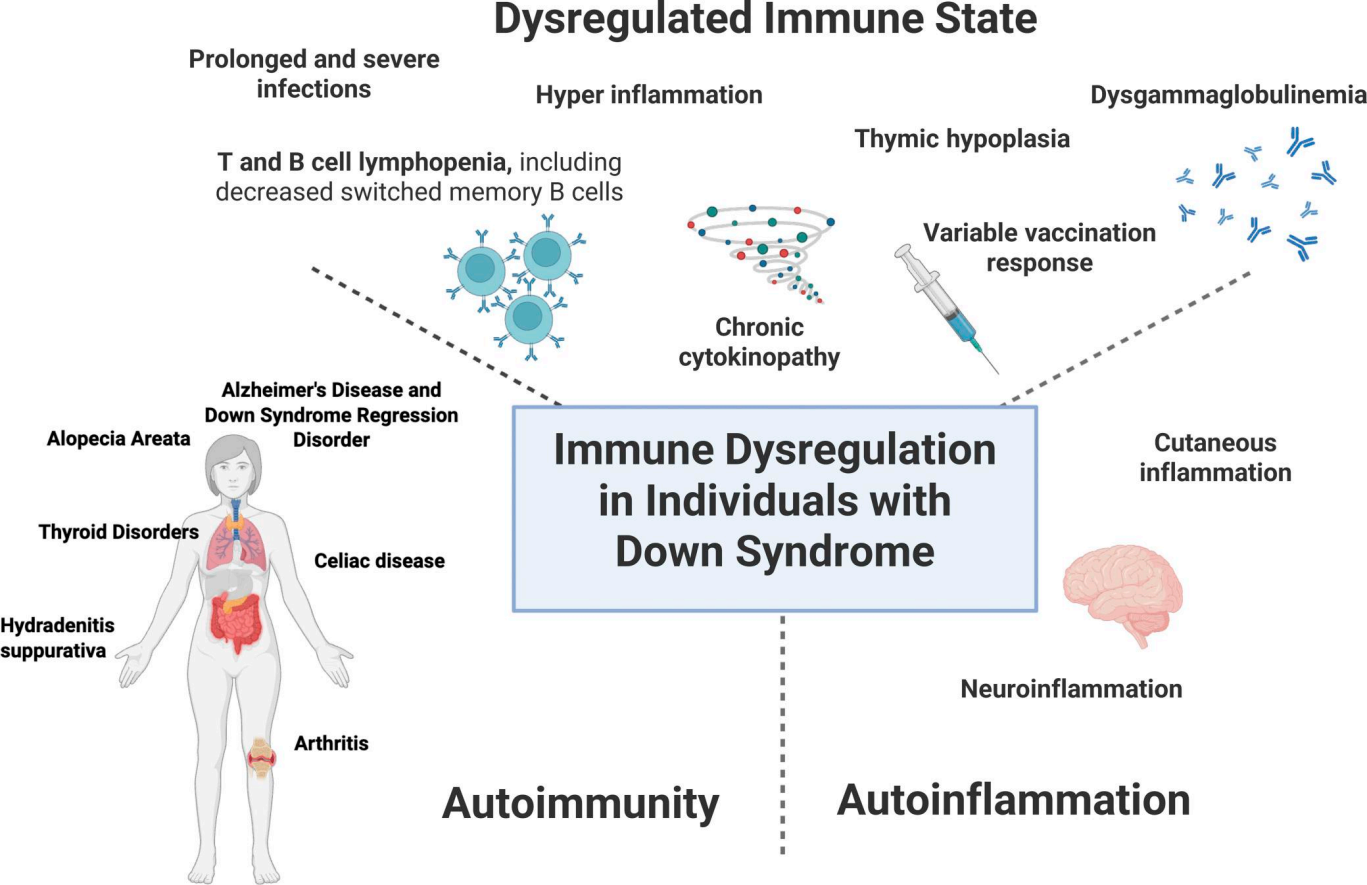
Schematic explanation of immune dysregulation in patients with DS.

Despite the mounting evidence of immune dysregulation in DS proven by consistent and reproducible immune abnormalities (Fig. 1), DS is not currently recognized in the human inborn errors of immunity classification by the International Union of Immunological Sciences Expert Committee as of the date of writing this article in 2025, which should be reconsidered (13). Such recognition may facilitate the development of more standardized and evidence-based investigation protocols for immunological testing and subsequent management strategies. Current guidelines for screening and management of pediatric and adult patients with DS also do not highlight aspects of immune dysfunction or provide recommendations for immunologic evaluation and surveillance (14, 15). DS societies and patient advocacy groups should also continue to raise awareness of the unique infectious challenges that patients with DS have.

In this review, we propose actionable insights (Table 1) into the clinical manifestations of immune deficiency and dysregulation associated with DS with the aim to help diminish the gap between emerging data and clinical practice for the practicing clinical immunologist. When to screen, when to use prophylaxis, when to intervene, and how aggressively to act are all key questions. Of note, the immune system’s role in the risk of malignancy is recognized in this population; however, this is beyond the scope of this article. Here within, we will describe real clinical cases to exemplify how we treat immune dysregulation in patients with DS. References are provided where appropriate, and the remainder expresses the opinion of us, the authors, through clinical experience and expertise.

**Table 1.** Recommended evaluation and management for the immunodeficiency, autoimmunity, and autoinflammation in the immune dysregulation of DS

Immunologic domain	Recommended evaluation	Management recommendations
Immunodeficiency	• Quantitative lymphocyte subsets (CD3, CD4, CD8, and CD19) for all DS patients once in early childhood or at initial referral with annual repeat if low counts, other immunologic laboratory abnormalities, or on treatment • Quantitative IgG, IgA, IgM, and IgE for all DS patients once in early childhood or at initial referral with annual repeat if low counts, other immunologic laboratory abnormalities, under age 4, or on treatment • Vaccine-specific antibody titers (ex. pneumococcal, measles, mumps, rubella, varicella, hepatitis B, tetanus, and diphtheria) at age 2 years or at initial referral if older • Consider B cell phenotyping if other immunologic laboratory abnormalities and considering treatment	**Vaccination** • Ensure complete age-appropriate vaccinations • Live vaccines generally safe but defer if CD4 <400/μl or CD8 <200/μl • RSV, Covid-19, and influenza recommended • Prefer conjugate pneumococcal vaccines; booster schedules for age >2 years per immunocompromised CDC guidance • Nonresponders may benefit from revaccination using conjugate pneumococcal formulations or shortened booster intervals **Treatment** • Timely and liberal treatment of infections • Low threshold for low-dose antibiotic prophylaxis (ex. azithromycin) for mild/moderate recurrent infections • Low threshold for immunoglobulin replacement in SPAD or CVID-like phenotypes (400–600 mg/kg/mo; target IgG trough ∼800–1,000 mg/dl) • Multidisciplinary care is crucial to optimize anatomic susceptibilities to infection **Monitoring** • Monitor complete blood cell counts, renal function, and liver function closely on immunoglobulin replacement • Recheck titers 4–8 wk after vaccination to assess response
Autoimmunity	• Follow established DS surveillance guidelines (thyroid routinely; symptom-based screening for diabetes, celiac) in collaboration with primary care physician • Baseline autoimmune serologies before IVIG initiation	**Treatment** • Multidisciplinary management with endocrinology, gastroenterology, rheumatology, neurology, psychiatry, and other specialties as clinically indicated • Immunomodulatory or immunosuppressive therapy when indicated **Monitoring** • Evaluate new systemic, neurologic, or behavioral symptoms promptly • Recommend heightened infection monitoring when on immunosuppression
Autoinflammation	• Regular comprehensive skin examinations • Neuroinflammatory screening and referral when cognitive or behavioral changes present	**Treatment** • Aggressive control of inflammatory skin disease to reduce infection risk • Escalation to biologics when standard therapies fail as clinically indicated • Consider JAK inhibitors for IFN-driven disease • Supportive care and infection-directed therapy as needed **Monitoring** • Consider trending inflammatory markers and selected cytokine studies

## Clinical vignette 1

A 6-year-old boy with DS, congenital heart disease previously not requiring surgery, and intellectual disability presented to immunology clinic for evaluation of recurrent infections. His past history included recurrent viral and bacterial infections, including otitis media, croup, pneumonia, pharyngeal abscess, folliculitis, and conjunctivitis. He had approximately two lifetime hospitalizations for respiratory failure from pneumonia requiring supplemental oxygen and was hospitalized for the one lifetime pharyngeal abscess. For the aforementioned infections, he would on average received antibiotics five to eight times per year. He would be diagnosed with otitis media, croup, pneumonia, folliculitis, and conjunctivitis multiple times per year. He had never been on antibiotic prophylaxis but did have myringotomy tubes placed. Labs showed mild hypogammaglobulinemia (IgM 18 mg/dl, IgG 499 mg/dl, IgA 42 mg/dl, and IgE 3 mg/dl), CD19 lymphopenia (79 cells/ml), CD8 lymphopenia (161 cells/ml), and reduced class-switched memory B cells. He had normal distribution of naïve, memory, and terminally differentiated effector memory T cells (TEMRA) T cell markers. Postvaccination vaccine-specific antibody responses revealed nearly absent pneumococcal titers despite boosters; absent measles, mumps, rubella, varicella, and hepatitis B titers; and preserved tetanus and diphtheria titers. Immunoglobulin replacement therapy was recommended to the family for a diagnosis of specific antibody deficiency (SPAD) at a starting dose of 400 mg/kg/mo by subcutaneous infusion. Subcutaneous infusion was recommended to decrease risks of side effects associated with intravenous administration. Family was offered the option of hyaluronidase-facilitated immunoglobulin at a dosing interval of 4 wk or non-hyaluronidase–facilitated immunoglobulin every 2 wk. Unfortunately, the patient’s family declined to start immunoglobulin. If the patient was started on immunoglobulin replacement, he would have been closely monitored every 6 mo to monitor complete blood cell counts, kidney function, liver function, immunoglobulin troughs, and clinical response in terms of frequency and severity of infections. If the patient clinically improved and was largely free of severe or frequency infections for 1–2 years, the family would have been offered a trial off immunoglobulin to see if sustained improvement. The patient is now 12 years of age and continues with a similar infectious history and similar immune profile (Table 2).

**Table 2.** Summary of three clinical vignettes exemplifying real-world cases of patients with DS and immunodeficiency, autoimmunity, and autoinflammation, respectively

Patient age	Other past medical history	Presenting issue	Laboratory and clinical findings	Diagnosis	Recommended treatment
Case #1 6-year-old male with DS	Congenital heart disease	Recurrent infections (otitis media, croup, pneumonia, pharyngeal abscess, folliculitis, and conjunctivitis)	Mild hypogammaglobulinemia (IgM 18 mg/dl; IgG 499 mg/dl; IgA 42 mg/dl; IgE 3 mg/dl); CD19 and CD8 lymphopenia; reduced class-switched memory B cells; absent pneumococcal, measles, mumps, rubella, varicella, and hepatitis B titers despite boosters; preserved tetanus and diphtheria titers	Specific antibody deficiency	Immunoglobulin replacement therapy
Case #2 25-year-old male with DS	N/A	Poor oral intake and dysphagia; anorexia and 50-lb unintentional weight loss; gait instability, cognitive slowing, social withdrawal, and fatigue	Imaging consistent with sialadenitis; autoimmune hypothyroidism and primary adrenal insufficiency; brain MRI showing mild ventriculomegaly, cerebellar hypoplasia, and basal-ganglia mineralization; normal Alzheimer’s biomarkers and vitamin B12	DSRD	Levothyroxine and corticosteroids; considering benzodiazepines, immunoglobulin, and JAK inhibition
Case #3 16-year-old female with DS	Hypothyroidism, obstructive sleep apnea, eruptive syringomas, seborrheic dermatitis, papular eczema, and alopecia areata	Progressive, treatment-refractory hidradenitis suppurativa with painful axillary abscesses requiring hospitalization	Persistent severe hidradenitis suppurativa flares despite adalimumab therapy, prolonged doxycycline, and multiple intralesional triamcinolone injections; required intravenous antibiotics and high-dose corticosteroids	Hidradenitis suppurativa in the context of DS-associated cutaneous inflammation	Transition from adalimumab to secukinumab

## Immunodeficiency in DS

Infection is the leading cause of mortality in patients with DS and an etiology for decreased quality of life in this population (3, 16, 17, 18). Clinically, this is represented by a bit of a paradox, where incidence of some infections is actually lower, but when infection occurs the outcomes are significantly worse as compared to the typical population. For instance, bacterial infection (pneumonia, sinusitis, otitis, conjunctivitis, and skin) and viral infections (influenza and SARS-Cov2), periodontal disease, acute respiratory distress syndrome, and life-threatening sepsis are more common in patients with DS (17, 19, 20, 21). However, patients with DS have lower incidence of strep pharyngitis, tonsillitis, sexually transmitted infections, mononucleosis, shingles, and intestinal infections (19). To manage these recurrent infections in DS, multidisciplinary care with other specialists is crucial to minimize the detrimental effects of the underlying anatomy in DS. For instance, the patient in the first clinical vignette was referred to otolaryngology for myringotomy tubes due to recurrent otitis, ophthalmology for guidance on increased tear duct circulation and lubrication to minimize conjunctivitis, pulmonology for airway clearance to prevent bronchiectasis, gastroenterology to minimize reflux symptoms contributing to recurrent infections, and pediatric dentistry for regular periodontal screenings. Occupational and physical therapy and dieticians are also important for improving hypotonia, strength, ability to cough, and optimize nutritional status. However, the immunologist is crucial to manage the intrinsic immunodeficiency in DS and should not be forgotten while other specialists manage the individual organs. Many academic medical centers have multidisciplinary clinics for patients with DS or recognized internal “experts” who have a particular interest in DS for referrals, and this works well for coordination and optimization of care. The immunodeficiency in DS is sometimes accompanied by lymphopenia, hypogammaglobulinemia, and/or poor postvaccination antibody responses. Antibiotic prophylaxis and/or replacement immunoglobulin can be utilized to help mitigate these infections in DS.

## Lymphopenia

Compromised cellular immunity with mild to moderate absolute lymphopenia is a frequent immunological finding in DS and encompasses at times reductions in T and almost uniformly in B cell populations (22, 23, 24, 25, 26, 27). The prevalence of lymphopenia in DS ranges in cohorts from 20% to 80%, a substantial amount higher than the rate of lymphopenia estimated in the general population (around 1%) (28).

T cell lymphopenia is explained by impaired thymocyte maturation and defective T cell maturation and thymic hypoplasia—with DS thymuses being smaller and more hypocellular leading to reduced thymic output (10, 29, 30, 31). T cell receptor excision circles as commonly accessed by newborn screen for severe combined immunodeficiency can be quite low in DS patients due to this lymphopenia, and patients may even screen positive on newborn screen (32, 33). However, it should also be noted that though naïve T cells are decreased, memory T cells are similar to age-matched controls, favoring intrinsic defect in T cells over early senescence (34). T cells in patients with DS have decreased function with laboratory studies showing reduced proliferation to mitogens, premature aging, and increased T cell exhaustion (30, 35, 36, 37).

B cell lymphopenia in DS is highlighted by low numbers of total, naive, and switched memory B cells, almost uniformly (24, 38, 39, 40). Whereas T cell levels in childhood may reach more normal levels over time, B cell lymphopenia persists and appears to be even more pronounced in adult life (24). However, while the B cell lymphopenia pattern is similar to those with common variable immunodeficiency, most patients with DS are not hypogammaglobulinemic, and it is not clear if the B cell lymphopenia is due to an intrinsic defect or decreased T lymphocyte help (41).

All patients with DS should thus have T and B cell levels quantitatively assessed by flow cytometry for CD3, CD4, CD8, and CD19 on initial evaluation by an immunologist. In our practice, we typically check these on initial referral and then repeat annually if quite low or the patient has other clear immunologic laboratory abnormalities or is started on treatment with prophylactic antibiotics or immunoglobulin. For the patient in the first clinical vignette, he is followed annually for repeat lymphocyte subset phenotype due to decreased counts. If the T cell counts are normal in addition to other laboratory values, they are only repeated if the patient’s infectious history significantly changes. In our experience, T cell numbers do not typically drastically decrease and often improve over time, so baseline reassuring values do not need to be followed closely. Quantification of naïve versus memory T cell counts may not always be helpful in clinical practice in patients with DS, though distribution between naïve and memory subsets can deviate from normal age-adjusted values. Conversely, B cell phenotyping in patients with DS is more clinically useful as low switch memory B cells can be a marker of impaired antibody responses. Recent thymic emigrants can also be decreased in patients with DS but are rarely clinically useful, as we know that the thymus is smaller with poorer output in DS. Patients with DS are not typically at risk for Omenn syndrome or oligoclonal T cell lymphocytosis. For that reason, monitoring T cell receptor repertoire is also not very useful in clinical practice for patients with DS. Routine T cell functional testing through mitogen-induced lymphocyte proliferation studies or lymphocyte proliferation to anti-CD3, anti-CD28, or anti-IL2 can be abnormal in patients with DS, though unlikely to be useful in routine clinical practice.

## Hypogammaglobulinemia

Despite B cell lymphopenia, panhypogammaglobulinemia is not a typical characteristic feature with many cohorts of DS patients describing normal, increased, or solely decreased levels of IgM (9, 22, 24, 39, 40, 42, 43, 44). While IgG may be normal, IgG subclass deficiency, specifically IgG4 deficiency has been implicated in patients with predisposition to infection, and it is debatable whether measuring this may assist in clinical assessment (45). Even with normal levels of immunoglobulins, there is increased susceptibility to severe infections, which may be related to an intrinsic B cell differentiation defect and impaired antibody maturation (38, 39, 42). Diagnosis of common variable immune deficiency (CVID) in patients with DS per CVID diagnostic criteria is rare, whereas SPAD is more common, like our first clinical vignette (46).

In our clinical practice, we evaluate all patients with DS with immunoglobulin quantification of IgG, IgA, IgM, and IgE. If evaluated under age 4, we evaluate this annually to ensure normal IgG levels have been gained after the expected nadir of infancy. Levels are then repeated as needed if there is a change in infectious history. However, most patients with DS have normal immunoglobulins despite clear immune dysfunction and normal immunoglobulin levels alone are not reassuring against immunodeficiency nor indication to not refer to immunology for these patients.

## Vaccine response

Vaccine responses in DS can vary, and it is common for DS patients to not appropriately respond to all vaccines. However, it is not common for patients with DS to not respond to all or most vaccines like the aforementioned patient. For instance, hepatitis B may be diminished compared to healthy controls (39, 47, 48, 49, 50, 51, 52, 53, 54). DS patients may also respond better to protein-conjugate pneumococcal vaccinations as compared to unconjugated polysaccharide pneumococcal vaccines (47, 48, 51, 53). Meningococcal vaccination responses may also be decreased (55). Vaccine responses in DS are also more likely to be lost over time (56). In our first clinical vignette, the patient responded to tetanus and diphtheria vaccinations but not others, which is consistent with an impaired response to vaccines, as tetanus and diphtheria are known among the common childhood vaccines to be the most immunogenic with highest seroconversion rate in healthy populations (57).

A comprehensive vaccination review is foundational. Patients with DS should receive all age-appropriate inactivated vaccines as per the standard American Academy of Pediatrics schedule (58). There are no contraindications to any vaccines intrinsic to patients with DS. Furthermore, we suggest an increased uptake of vaccination against respiratory syncytial virus (RSV), Covid-19, and influenza, as individuals with DS are at increased risk of morbidity and mortality from these lower respiratory viral infections (4, 8, 59, 60, 61, 62, 63). We recommend specific vaccine titers be assessed in all DS patients around 2–4 years of age after completing the primary childhood vaccine series and patients be revaccinated if poor titers are evident, especially for *Streptococcus pneumoniae*. Some have suggested tailored vaccination schedules to increase the number of switched memory B cells to improve protection among patients with DS (39, 64). We support this and believe that DS patients benefit from booster pneumococcal administration for immunocompromised patients per Centers for Disease Control (CDC) criteria after the age of 2 with preference given to the conjugate form of the vaccine. Postvaccination serology can be rechecked 4–8 wk later to assess response (65). Nonresponders may benefit from revaccination using conjugate pneumococcal formulations or shortened booster intervals. Live attenuated vaccines are generally safe in DS but should be deferred in those with significant T cell lymphopenia (CD4 <400 cells/μl or CD8 <200 cell/μl), similar to those guidelines recommended for DiGeorge syndrome (66). Though extrapolating these guidelines should be interpreted with caution and done so by using age-adjusted reference intervals. Finally, we hope that studies addressing the quality of vaccine response be studied, as the mere presence of antibodies, if low affinity, may not mean true protection from diseases despite “normal” titers.

## Antibiotic prophylaxis and immunoglobulin replacement

For individuals with recurrent sinopulmonary infections or established bronchiectasis, low-dose antimicrobial prophylaxis may reduce infection frequency and airway inflammation. Prophylaxis should be tailored to culture results, tolerance, and risk of resistance. We favor azithromycin for prophylaxis in the DS population extrapolating on prior studies in non-DS patients with primary antibody deficiency and utilizing recent data on a small cohort of patients with DS (67, 68). However, in our experience, antibiotic prophylaxis is not as efficacious as immunoglobulin replacement, and we typically reserve it for cases with mild infections or where families are hesitant to start immunoglobulin replacement. However, there are no head to head trials comparing immunoglobulin replacement to antibiotic prophylaxis in patients with DS. We also recognize that immunoglobulin replacement is not available everywhere and the cost for antibiotic prophylaxis is much less. As most patients with DS will have recurrent sinopulmonary infections in the first years of life and then this tends to improve, antimicrobial prophylaxis may be preferred over immunoglobulin replacement for younger patients with DS such as those under 6 years. Immunoglobulin replacement can then be considered for those with DS who persistently have infections past that age and/or antibiotics are not effective.

Medical providers should also, however, keep in mind that it is well known that patients with DS have a weakened innate immune system in addition to the adaptive immune system. Even when the adaptive immune system in DS improves, there remains a more severe response to infection. Neutrophil chemotaxis is impaired in DS and may explain higher rates of mortality in sepsis in DS (17, 69). Given this, for patients with DS who are actively infected, we are more liberal in treating with antibiotics, as their innate immune system may not be able to clear an infection as readily on its own.

Intravenous immunoglobulin (IVIG) or subcutaneous immunoglobulin replacement should be considered in patients meeting criteria for SPAD or CVID-like phenotypes—particularly when recurrent or severe infections persist despite optimal vaccination and antibiotic prophylaxis. Typical dosing is the same as for other replacement regimens for inborn errors of immunity (400–600 mg/kg/mo), adjusted to maintain adequate IgG troughs (800–1,000 mg/dl) that correlate with clinical response. Patients often report decreased infection burden and improved quality of life within 6 mo of initiation. However, as patients with DS have immunodeficiency beyond the humoral defect and other anatomic risk factors for infection, replacement immunoglobulin may not prevent all of their infections. Ongoing reassessment is necessary to determine the continued need for therapy.

We believe immunologists should have a low threshold to initiate immunoglobulin replacement therapy and/or prophylactic antibiotics in patients with DS. Even in DS patients with borderline laboratory findings to meet criteria for SPAD or CVID, we know that there are more immunological abnormalities than is identified by routine immunological testing, and we know that these patients have increased structural and anatomic abnormalities contributing to severe recurrent infections. Furthermore, immunoglobulin likely has a greater role than just humoral replacement in this population. We know that immunoglobulin can decrease autoimmunity and autoinflammation that patients with DS commonly have (70). Therefore, we strongly believe in optimizing their immune function through replacement immunoglobulin when feasible. However, we do not recommend committing patients with DS to lifelong treatment with antibiotic prophylaxis or immunoglobulin replacement when either is started. The immune system in DS can wax and wane over time, and trial periods off therapy are recommended to see if there is a continued need.

There are some additional considerations for replacement immunoglobulin in patients with DS. Due to the high incidence of intellectual disability, autism, and other neuropsychiatric diagnoses in DS, subcutaneous immunoglobulin administration is sometimes logistically difficult for behavioral reasons, and intravenous may be preferred by the family (71). However, another important consideration is that many patients with DS have congenital heart disease and subcutaneous replacement is often preferred in congenital heart disease to minimize risk of fluid overload and minimize immunoglobulin losses if protein losses. Monitoring for cytopenias, kidney function, and liver function is routine in all patients on immunoglobulin replacement but is especially important in those with DS who are at high risk for anemia, chronic kidney disease, and metabolic dysfunction–associated steatotic liver disease (26, 72, 73, 74).

## Clinical vignette 2

A 25-year-old man with DS presented with 3 days of poor oral intake and dysphagia following several months of progressive anorexia and 50-lb unintentional weight loss. Over the preceding month, he developed gait instability, cognitive slowing, marked social withdrawal, and fatigue. At prior baseline, he was sociable, active, independent, and fully ambulatory. He was admitted for dehydration. Imaging revealed asymmetric enhancement of the left parotid and bilateral submandibular glands, consistent with sialadenitis. Endocrine evaluation confirmed autoimmune hypothyroidism and primary adrenal insufficiency. He received levothyroxine, steroids, and gastrostomy-tube placement. Brain magnetic resonance imaging (MRI) showed mild ventriculomegaly, cerebellar hypoplasia, and basal-ganglia mineralization. Alzheimer’s biomarkers and vitamin B12 were normal. In the absence of structural or infectious pathology, the presentation was consistent with autoimmune-mediated DS Regression Disorder (DSRD). Benzodiazepines, IVIG, and Janus kinase (JAK) inhibition with tofacitinib were considered for treatment. The patient started to have some spontaneous improvement, and the family wanted to defer treatment at this time.

## Autoimmunity in DS

Autoimmunity is widely prevalent in DS and among the most well-known immunologic manifestations. It is clinically diverse, including thyroid disease, type 1 diabetes mellitus, celiac disease, alopecia areata, DS-associated arthritis, and neuroinflammatory conditions such as DSRD and Alzheimer’s disease (75).

The autoimmunity in DS is influenced by several factors such as impaired thymic T cell production, increased inflammatory cytokines, elevated IFN signaling, chronic T cell overactivation, and reduced T regulatory cell function with effector T cells resistance to T regulatory–mediated suppression (76, 77). T regulatory cells are numerically increased and can be functionally impaired. Individuals with DS can have expansion of CD11c^+^ T-bet^high^ CD21^low^ B cells that given their extrafollicular origin likely contribute to autoantibody production (77). We know that patients with autoimmune regulator (AIRE) deficiency present with autoimmunity. AIRE is encoded on chromosome 21, and surprisingly, in individuals with DS who have an extra chromosome 21, there is reduced and not increased AIRE expression, and age-dependent expression decline after infancy, impairing thymic deletion of autoreactive T cells, thus promoting autoimmunity (78, 79).

Collectively, these findings describe a chronic pro-inflammatory, self-reactive immune state that underlies multiorgan autoimmunity in DS. In the second clinical vignette, such dysregulation likely accounted for his autoimmune hypothyroidism, adrenal insufficiency, and neurobehavioral regression-features consistent with autoimmune DSRD.

Individuals with DS require targeted medical surveillance due to their elevated risk for specific autoimmune comorbidities. Routine thyroid screening is strongly recommended by both the American Academy of Pediatrics and the Global Down Syndrome Foundation guidelines (14, 15). Additionally, individuals with DS have an increased risk of celiac disease and type 1 diabetes, for which current recommendations emphasize symptom-based screening to ensure timely detection and management done in collaboration with endocrinology and gastroenterology (14, 15). Inflammatory arthritis may require rheumatologic input and be treatment resistant (80). DS-associated arthritis can present differently from non-DS patients: being more likely to present with multiple joints, have delayed diagnosis, and be treatment resistant.

In our clinical practice, it is essential that patients with DS have a medical home and a primary care provider who they see regularly and is familiar with the routine screening guidelines for autoimmunity in DS (14, 15). Clinical immunologists should also be aware and refer appropriately if new symptoms arise in their patients. This autoimmunity in DS may require immunomodulatory or immunosuppressive medications, and while not contraindicated in this population, there is poor data on most of these agents in DS, and there may be immunological downstream effects that we cannot predict. Patients with DS on these agents require vigilant infection monitoring due to baseline immune vulnerability. It should also be noted that patients with DS who are started on immunoglobulin should have baseline autoimmune titers drawn prior to starting immunoglobulin.

Finally, in the era of chimeric antigen receptor T-cell therapy (CAR-T) cells targeting CD19-positive cells, while counterintuitive for infections, therapy like this, especially with immunoglobulin replacement present, may truly transform the landscape of autoimmunity in individuals with DS. They deserve a clinical trial dedicated to this type of investigation.

## DSRD

DSRD refers to an acute neurocognitive decline occurring in individuals with DS and is increasingly linked to an underlying autoimmune etiology (81, 82). Several studies highlight the potential benefit of immunomodulatory therapy. Studies report marked improvement with IVIG even in the absence of detectable autoantibodies (83, 84). Santoro et al. (2022) showed remarkably high response rates (88–92%) to IVIG in his study that enrolled 72 DSRD patient treatments and found the greatest IVIG efficacy in patients with cerebrospinal fluid inflammation (81). Corticosteroids remain first-line therapy in many neuroimmunologic disorders but have shown variable benefit in DSRD (30–50%), possibly due to heterogeneous dosing and comorbidities. For patients with confirmed neuroinflammation, mycophenolate or azathioprine may be used as second-line agents (81).

Other interventions may be useful when psychiatric or noninflammatory mechanisms predominate. Benzodiazepines and electroconvulsive therapy have shown response rates around 70–75%, particularly in individuals without neurodiagnostic abnormalities, suggesting that psychiatric and immune-mediated subtypes may coexist within the DSRD spectrum (81).

Emerging data implicate IFN-JAK/STAT hyperactivation in DS (12, 85, 86). The JAK inhibitor tofacitinib has shown promise in reducing IFN signaling and improving cognition and behavior in DS-related autoimmune disease (87), and there is an ongoing clinical trial studying JAK inhibitors in DSRD (NCT05662228). Brain imaging findings in DSRD are often abnormal and similar to other interferonopathies, which further supports the potential use of JAK inhibitors in DSRD (88). CAR-T cells also may have a role in treatment of DSRD. However, using JAK inhibitors and other immunomodulatory medications requires careful monitoring.

Despite growing recognition of DSRD as an immune-mediated entity, management remains unclear due to overlapping specialty domains. Immunologists, neurologists, psychiatrists, rheumatologists, and geneticists may differ on who should lead immunotherapy decisions to treat DSRD, often resulting in treatment delays—which occurred in our case. In multidisciplinary care for treating DSRD, we believe that the immunologist can prescribe treatment but recognize that many immunologists may feel uncomfortable prescribing these immunomodulatory medications. This is why coordination of care among specialists is essential. There are no clear guidelines for management of DSRD that clinicians can reference, and globally there is greatly decreased awareness of DSRD as an entity at all. This leads many patients with DSRD to not be treated and significantly decreased quality of life for the patient and caregivers. This case underscores the need for clear consensus guidelines and coordinated multidisciplinary care to define diagnostic pathways, determine therapeutic candidacy, and optimize outcomes for individuals with autoimmune forms of DSRD.

## Alzheimer’s disease

DS is also associated with neuroinflammatory processes that contribute significantly to the early onset of Alzheimer’s disease, a well-documented phenomenon in adults with DS over the age of 40 (89). This is distinct from DSRD, which typically has an onset much younger and more abruptly. As a result, routine screening for Alzheimer’s disease beginning at age 40 is strongly recommended (14). However, it is not quite clear what is the best treatment option for Alzheimer’s disease in DS and what is actually effective (90).

## Clinical vignette 3

A 16-year-old adolescent with DS has a 3-year history of hidradenitis suppurativa, eruptive syringomas, seborrheic dermatitis, papular eczema, and alopecia areata. Other comorbidities include hypothyroidism since infancy and obstructive sleep apnea. Her hidradenitis suppurativa has progressed over the past year, prompting initiation of adalimumab for disease control, an extended course of doxycycline, and multiple intralesional triamcinolone injections. 6 mo later, she continues to have severe axillary flares with abscesses, resulting in severe pain and loss of function, necessitating hospitalization, intravenous antibiotics, and high-dose corticosteroids. Given suboptimal disease control and ongoing morbidity, she is being transitioned from adalimumab to secukinumab as part of an escalated biologic strategy. JAK inhibition could also be considered to treat this patient’s autoinflammation as off-label therapy.

## Cutaneous inflammation in DS

There are many dermatologic manifestations associated with DS (91, 92). Folliculitis, seborrheic dermatitis, hidradenitis suppurativa, eczema, alopecia areata, xerosis, syringomas, onychomycosis, and psoriasis are just some of the common skin conditions seen in these patients. In our clinical practice, we believe comprehensive regular skin exams are essential for patients with DS by all of their medical providers, and there should be a low threshold to refer patients with DS and common dermatological findings to a dermatology specialist to establish care, as there is a high likelihood of other dermatological findings occurring in the future for these patients. Adequate control of these underlying skin conditions is important as lack of control increases the risk of cutaneous infection. We do want to specifically mention that hidradenitis suppurativa is particularly common in patients with DS and often misdiagnosed as bacterial abscesses. Hidradenitis suppurativa needs to be recognized early in patients with DS and have the autoinflammation treated (93).

## Autoinflammation in DS

This third clinical vignette highlights the predisposition to heightened inflammation seen in individuals with DS. DS is increasingly recognized as a chronic cytokinopathy in which individuals exhibit stable elevations in multiple pro-inflammatory cytokines. The presence of four of the six IFN receptor genes on chromosome 21 is strongly implicated in amplified inflammatory phenotypes (12, 86, 94, 95, 96). The resulting hyperresponsive IFN pathway produces a mixed type I/II interferonopathy phenotype, contributing to exaggerated inflammatory and cytokine-driven manifestations in affected individuals (12). Therefore, JAK inhibitors have been studied to decrease this autoinflammation driven by interferonopathy in DS patients (97, *Preprint*, 98). While JAK inhibitors are promising for treating the immune dysregulation in DS, a study comparing a patient on a JAK inhibitor with DS and STAT1 gain of function compared to a patient only with STAT1 gain of function on a JAK inhibitor suggests that there remains persistent inflammation in DS even with a JAK inhibitor (99). Currently a clinical trial for severe atopy using the next generation JAK inhibitor, abrocitinib, is being investigated solely in individuals with DS (NCT07242638). This is important as individuals with DS may require different dosing; the start of therapy need not be benchmarked to the typical population, and unfortunately, still, most often, individuals with DS are being excluded from clinical trials.

The immune dysregulation in the third clinical vignette is demonstrated by the treatment resistant hidradenitis suppurativa and the need for greater immunosuppression, perhaps to control the autoinflammation in DS. However, the autoinflammation in DS in other patients can also include exaggerated responses to infection and a hemophagocytic lymphohistiocytosis-like phenotype (8, 100, 101, 102, 103). The autoinflammation in DS is more likely to be driven by IFN than natural killer cell dysfunction in DS, as there is a paucity of data demonstrating natural killer cell dysfunction in DS (104). Therefore, we do not find it clinically useful to periodically examine natural killer cell lymphocyte phenotyping or functional studies in DS patients.

In clinical practice, we find it sometimes useful to trend cytokine panels, IFN γ signature, or specific markers of IFN γ production like IL-18 and CXCL9 plasma/serum levels in DS patients with autoinflammation. This can help guide treatment, monitor response to treatment, and help differentiate infection from autoinflammation in these patients. However, cytokine testing is expensive, not widely available, not always reproducible, and has poor sensitivity and specificity. In the context of treatment, use of emapalumab likely needs more exploration as does use of anifrolumab, both of which are likely to help individuals with DS. However, there is limited data overall for the use of immunomodulator medications in DS as patients with DS are often excluded from clinical trials.

## Conclusions

Despite DS being so common, the immune dysregulation in DS remains underrecognized and understudied. Most patients with DS never see an immunologist. Many non-immunology providers caring for patients with DS do not understand the role of the immune system in DS and do not appreciate the unique differences in care that their medical conditions require. In stark contrast, caregivers of patients with DS are often tangibly aware of the differences between those with DS and those without. They can appreciate the differences in response and duration of infections and how common diagnoses often given to patients with DS, such as asthma and eczema, are different in these patients compared to others. A strong medical home with pediatricians and internists aware of the immune dysregulation in DS for patients with DS is essential. There needs to be global guidelines and increased awareness and referral patterns from this strong medical home.

We also recognize that many of the recommendations made here within may not be applicable on a global scale and in resource-poor settings. When limited resources are available, we do not want to focus on the lymphocyte phenotyping, which may not be of clinical significance or change management. We would also not focus on total immunoglobulins, which are usually normal in DS. We would, however, focus on vaccine responses, which can be easily optimized through vaccine boosters. Immune evaluation can also be tailored based on the individual patient’s clinical history, with more detailed immunological evaluation for those presenting with concerning symptoms as opposed to screening labs for all patients with DS, as labs are more likely to be abnormal with a concerning clinical history (105).

We also recognize that immunomodulatory agents and immunoglobulin are not available everywhere and are quite costly. It is also even more difficult to obtain these agents even when available in the absence of approvals and guidelines demonstrating their use in DS specifically. Global effort should be made to support specific clinical trials focused on patients with DS or at least include patients with DS for medications such as JAK inhibitors. We also need data looking at immunoglobulin replacement in patients with DS above 6 years of age verses not replacing in terms of impact on infections, inflammation, autoimmunity, and quality of life. Furthermore, we need vaccine response studies systemically performed in patients with DS that evaluate the immune system in DS’s response to vaccines beyond protective titers.

As exemplified in the aforementioned cases, the immune dysregulation in DS is a spectrum and can be subtle or profound and manifest itself through a variety of clinical features in immunodeficiency, autoimmunity, and autoinflammation. More research is needed to phenotype patients with DS so we can predict how this immune dysregulation will present. Nonetheless, the clinical immunologist’s role in managing the immune dysregulation in this vulnerable population is essential.
